# Linking deprivation in early childhood with academic performance in middle adolescence through cognitive ability in middle childhood: Nuance by specific cognitive component and heterogeneity by child negative emotionality

**DOI:** 10.1017/S0954579425100813

**Published:** 2025-10-24

**Authors:** Shaofan Wang, Nan Zhou, Hongjian Cao, Xiuyun Lin

**Affiliations:** 1 Department of Psychology, The University of Hong Kong, Hong Kong SAR, China; 2 Research Hub of Population Studies, The University of Hong Kong, Hong Kong SAR, China; 3 Faculty of Education, University of Macau, Macau SAR, China; 4 Institute of Developmental Psychology, Faculty of Psychology, Beijing Normal University, Beijing, China; 5 Beijing Key Laboratory of Applied Experimental Psychology, Beijing Normal University, Beijing, China

**Keywords:** Early Deprivation, Academic Performance, Cognitive Ability, Negative Emotionality, Developmental Cascades

## Abstract

Early deprivation holds far-reaching implications for academic performance in adolescence. Yet, the implicated cascading mechanisms remain under-delineated, and little is known about why children may display diverse patterns of cognitive development. To address such gaps, we leveraged long-term longitudinal data derived from the Future of Families and Child Wellbeing Study (*n* = 2,085). Results indicated that early deprivation (age 3, caregivers’ reports and observers’ ratings; controlling for early threat and unpredictability) was negatively associated with adolescent academic performance (age 15, adolescents’ reports) indirectly through a negative association with cognitive ability in middle childhood (age 9, standardized tests). Furthermore, such an indirect effect was less pronounced among children with higher (versus lower) negative emotionality (age 1, mothers’ ratings), given that the negative link between early deprivation and subsequent cognitive ability was weaker among children with higher (versus lower) negative emotionality. Breaking down cognitive ability into sub-components (i.e., working memory, language ability, reading comprehension, and problem-solving), both language ability and applied problem-solving were involved in the deprivation-emotionality interaction. These findings highlight the critical role of cognitive ability in accounting for the long-term academic consequences of early deprivation and the key role of negative emotionality in shaping heterogeneity in such pathways.

## Introduction

The developmental consequences of early deprivation exposure, primarily exemplified by parental neglect, parental absence, and material scarcity (Brooks-Gunn & Duncan, 1997; Ellis et al., 2022; Usacheva et al., 2022), have long been a focus of scientific research, intervention practice, policy-making, and social welfare (James et al., 2021; Reichman et al., 2001). This line of research can be traced back to the earlier influential work on family poverty (Brooks-Gunn & Duncan, 1997; Brooks-Gunn et al., 2021; Duncan & Brooks-Gunn, 2000) and studies adopting the cumulative risk perspective (Evans, 2004; Evans & Kim, 2007; Evans et al., 2013; Sameroff, 1998; Sameroff et al., 1987a). Despite the well-recognized comprehensiveness of such developmental consequences, we focus specifically on academic performance during adolescence as an outcome of interest in the present study, in light of its significant and lasting impacts across a wide range of domains over the life course, including higher education prospects (Tomasik et al., 2019), career success (Goering et al., 2023), and financial well-being (French et al., 2015). Studies consistently indicate that experiences of early adversities, especially deprivation-related ones (e.g., emotional and physical neglect and material hardship; Phillips et al., 2023), predict subsequent maladaptation in the academic domain (e.g., subpar GPAs, grade retention, and school dropout) across various life periods (Edmunds, 2020). However, the mechanisms implicated in the developmental cascades linking early deprivation with subsequent academic performance in adolescence remain underexplored.

Notably, beyond the independent and cumulative risk approaches (Evans et al., 2013; Sameroff et al., 1987b), research grounded in the later-developed Dimensional Models of Adversity (McLaughlin et al., 2021) suggests that early deprivation, as compared to threat and unpredictability of early life, appears to be more relevant and closely related to the subsequent development of cognitive ability (Machlin et al., 2025; Phillips et al., 2023; Usacheva et al., 2022), which is a key predictor of academic performance (Peng & Kievit, 2020). Accordingly, this study seeks to investigate whether cognitive ability as a potential mediating mechanism may account for the link between early deprivation and subsequent academic performance in adolescence. In addition, research has also suggested that early adversity may shape cognitive development in a *domain-specific* manner. Approaching cognitive ability as a broad, undifferentiated construct without specifying the sub-components would obscure important distinctions (Frankenhuis & de Weerth, 2013; Frankenhuis et al., 2016, 2020; Young et al., 2024). Given the multidimensional nature of cognitive ability, we further aim to obtain a more nuanced understanding of the role of cognitive ability by breaking it down into specific components when testing its potential roles in explaining the link between early deprivation and later academic performance in adolescence.

Furthermore, the theory of individual–environment interaction posits that children are not passive recipients of environmental influences (Sameroff, 2009). Children’s personal characteristics, such as temperament, likely shape the developmental implications of environmental influences, including early adverse ones (Hartz & Williford, 2015). Notably, not all children who experience early deprivation would necessarily develop subsequent cognitive deficits or poor academic performance (Collet et al., 2024; Wang et al., 2017), which highlights the necessity of identifying conditioning intrapersonal factors in such associations. Among those, negative emotionality, a temperamental predisposition to experience unpleasant affective states, such as irritability, fussiness, distress, and anger (Belsky et al., 1991; Slagt et al., 2016; Wang et al., 2017; Zhang et al., 2022), merits special attention, because research has extensively demonstrated that children with varied levels of negative emotionality differ in their vulnerability and/or even plasticity to the influences of adverse environments (Collet et al., 2024; Slagt et al., 2016; Suor et al., 2017). Therefore, another aim of the current study is to test the potential role of children’s negative emotionality temperament in altering the consequences of early deprived environments for later cognitive ability.

Methodologically, research on early adversity remains sparse using long-term longitudinal data collected from early childhood through adolescence. The existing ones usually rely on retrospective reports of childhood adversities many years later, which raises validity concerns as memories are prone to distortion and selective recall (Berg et al., 2020). To address this gap, this study leveraged valuable *prospective, multiple-method, and multiple-informant data* that extended from early childhood (age 3) through middle adolescence (age 15) among a large cohort of American children born from 1998 to 2000, to: (a) test a potential developmental cascade from deprivation in early childhood (age 3, caregivers’ reports and observers’ ratings) to academic performance in middle adolescence (age 15, adolescents’ reports) through cognitive ability in middle childhood (age 9, assessed with standardized psychological tests); (b) examine the potential moderating role of child negative emotionality (age 1, mothers’ ratings) in the link between early deprivation and later cognitive ability and academic performance; as well as (c) reveal the subtle nuance that has been largely obscured in prior research for the cognitive consequences of early deprivation in the mediating and moderating effects by disaggregating the broad cognitive ability into its specific components.

### The long shadow of early deprivation on subsequent academic performance

Academic performance in adolescence, which is often indicated by grades in various subjects (Edmunds, 2020; Lurie et al., 2023; Oeri & Roebers, 2022), has long been a child outcome of great research interest, given its comprehensive and enduring effects on adaptations across various domains throughout the lifespan, including prospects for higher education, professional achievements, and financial stability (French et al., 2015; Goering et al., 2023; Tomasik et al., 2019). Early deprivation refers to a lack of appropriate cognitive, social, and emotional inputs during the first few years of life, such as experiences of neglect, insufficient cognitive stimulation, and insecure access to basic life necessities (McLaughlin & Sheridan, 2016). Accordingly, early deprivation has often been indicated by limited cognitive, emotional, and material inputs in research (Lambert et al., 2017). Prior studies based on samples from different culture contexts and various historical times have consistently demonstrated the negative links between early deprivation-related adversities and later academic performance of children and adolescents (Oeri & Roebers, 2022; Qu et al., 2024; Slade & Wissow, 2007), especially the extensive and influential research on the cognitive and academic consequences of poverty (Brooks-Gunn & Duncan, 1997; Duncan & Brooks-Gunn, 2000; Duncan et al., 1994; Liang et al., 2024; Ryan et al., 2006).

Notably, to identify the unique impact of deprivation, it is important to control for other forms of adversities (i.e., early life threat and unpredictability) in analyses (e.g., Phillips et al., 2023; Wang et al., 2024), as suggested by the Dimensional Models of Adversity (McLaughlin et al., 2021). Early life threat can be conceptualized as adversities during early years of life that would pose the risk of physical or psychological harm to an individual (McLaughlin & Sheridan, 2016), such as experiencing and/or witnessing physical, sexual, and/or emotional abuse (Miller et al., 2021). Early life unpredictability represents the stochastic and unstable characteristics of early living environments (McLaughlin et al., 2021), which can be indicated by family transitions (e.g., parents’ divorce/separation/remarriage), fluctuations in family economic conditions, as well as residential instability (Doom et al., 2023; Usacheva et al., 2022). Research showing the unique academic consequences of early deprivation is still sparse. In a sample of 408 children (46.3% female) aged 10–13 from the Seattle area, Lurie and colleagues (2023) found negative associations between exposure to deprivation-related early-life adversities (e.g., physical and emotional neglect) and academic performance, controlling for the co-occurring threat-relevant early-life adversities (e.g., physical and emotional abuse). More efforts are still needed to systematically examine the independent, unique consequences of early deprivation exposure for academic performance above and beyond the effects of early life threat and unpredictability.

### A potential developmental cascade through cognitive ability

While the negative associations between early deprivation and later academic outcomes have been widely demonstrated, making further sense of such links increasingly hinges on examinations that aim to elucidate the underlying explanatory mechanisms. According to Sheridan and McLaughlin’s work (2016), exposure to deprived environments (e.g., parental emotional neglect) would be closely associated with later deficits in cognitive control, which are further linked to educational outcomes in school. This implies that cognitive ability may serve as a crucial mechanism linking experiences of early deprivation with later academic performance, where cognitive ability refers to an individual’s capacity to handle cognitive tasks, such as processing information, memory, learning, reasoning, and problem-solving (Grotzinger et al., 2019).

Early deprivation exposure is uniquely and negatively associated with overall levels and specific markers of subsequent cognitive ability, such as language development and executive functioning (Merz et al., 2013; Vogel et al., 2021; Young et al., 2024). The Dimensional Model of Adversity (McLaughlin et al., 2021) and the empirical research grounded in this model suggest that early deprivation, compared to threat and unpredictability, seems to be more pertinent to child cognitive development (Phillips et al., 2023; Usacheva et al., 2022). For example, by following 3,253 ethnically diverse children and their caregivers from low socioeconomic status families in the USA, Usacheva et al. (2022) found that early life deprivation – distinct from threat or unpredictability – at age three is specifically linked to cognitive ability around age five. Moreover, deprivation also seems to interfere with higher-order cognitive functions above and beyond the influences of threat-based adversities (Lambert et al., 2017; Miller et al., 2021). In a sample of US preschool children with greater socio-demographic risk, early deprivation, but not unpredictability (when included in the same model), was uniquely associated with diminished preschool executive control (Phillips et al., 2023). In addition, a recent meta-analysis also indicated that the links of deprivation with executive functions tended to be stronger than those for threat (Johnson et al., 2021).

Further, deficits in cognitive ability likely contribute to poor academic performance across the school years (Oeri & Roebers, 2022; Vogel et al., 2021). Disrupted cognitive development due to early deprivation, such as limited working memory capacity and inadequate problem-solving skills, likely heightens children’s difficulties in accomplishing various learning tasks during high school years (Blums et al., 2017; Merz et al., 2013), resulting in suboptimal academic performance, such as poor grades in various subjects. Likewise, research has also yielded evidence demonstrating that hindered neurocognitive development could account for the negative association of early deprivation with subsequent academic performance (Miller et al., 2021; Sheridan & McLaughlin, 2016).

Notably, given the multifaceted nature of cognitive ability, the subtle, domain-specific nuance still awaits being more systematically revealed for the cognitive consequences of early deprivation. An emerging body of research has indeed suggested that different components of cognitive ability may be differentially influenced by early experiences of deprivation (Frankenhuis & de Weerth, 2013; Frankenhuis et al., 2016, 2020; Young et al., 2024). For instance, among 8–17-year-old children who were adopted from psychosocially-depriving Russian institutions after 14 months of age and before 9 months of age, Merz and colleagues (2013) found that adolescents who were adopted after 14 months old (i.e., longer exposure to deprived environment) exhibited lower vocabulary skills and higher levels of attention issues compared to those who were adopted before 9 months (i.e., shorter early deprivation adversity). However, there were no differences between the two groups in nonverbal reasoning (Merz et al., 2013). They also found that attention and vocabulary skills played a mediating role in the link between early psychosocial deprivation and the utilization of educational support services. The negative predictions from early deprivation adversity to subsequent language skills and passage comprehension have also been observed in other studies after adjusting for the impact of threat-related adversities (Miller et al., 2021).

Besides, it seems that some cognitive components might not be significantly affected or even enhanced by early deprivation (Young et al., 2024). In a sample of Nigerian youth who lived in institutional homes and foster families, Nweze et al. (2021) found that youth with deprivation exposure did not differ from youth without deprivation experiences in their performance on set-shifting and inhibition tasks. Interestingly, the deprived group performed better than the nondeprived group in the working memory task (Nweze et al., 2021), which somewhat questioned the dominant view that early adverse rearing environments, especially deprivation, would necessarily impair child cognitive development. Notably, such seemingly “counterintuitive” patterns align with an emerging evolutionary model arguing that individuals exposed to early-life stress may demonstrate enhanced cognitive abilities that reflect their adaptations to harsh environments (Frankenhuis et al., 2016, 2020). In like manner, in a sample of 201 mother–child dyads living in a US Northeastern metropolitan area, Suor et al. (2017) found that exposure to environmental deprivation-based harshness (indicated by earned annual household income and maternal disengagement) at age 2 predicted worse visual problem-solving but better reward-oriented problem-solving at age 4, highlighting the necessity to consider how the cognitive consequences of early deprivation may vary as a function of cognitive ability components/domains.

### The potential moderating role of child negative emotionality

Even among children raised in the most precarious early environments, substantial variations in their subsequent cognitive abilities and educational achievements are evident (Young et al., 2024), which suggests that while some children are severely affected, others are still resilient and even able to excel in later development regardless of early adversities (Frankenhuis & Nettle, 2020; Frankenhuis et al., 2020). Hence, identifying the factors that may contribute to child vulnerability and plasticity to early adversities is crucial. Amongst the numerous potential factors, child temperamental characteristics (Slagt et al., 2016), especially the “difficult” ones such as negative emotionality (Suor et al., 2017; Zhang et al., 2022), merit special attention, given the ample evidence supporting the interaction between temperamental characteristics and environmental influences in predicting child development in both educational (Collet et al., 2024; Wang et al., 2017) and cognitive (Cha, 2018; Suor et al., 2017) domains.

As a core aspect of challenging temperament, children with high negative emotionality tend to display frequent, intense, or lasting episodes of anger, sadness, and frustration, especially under stressful circumstances (Slagt et al., 2016; Wang et al., 2017; Zhang et al., 2022). Its interactions with environmental influences in predicting child cognitive outcomes have been widely evidenced (Cha, 2018; Suor et al., 2017). More importantly, in response to early deprivation, children with different levels of negative emotionality appear to demonstrate various patterns of cognitive development (in hindered, immune, or even enhanced fashions).

Some perspectives have been proposed for these patterns. Classically, the diathesis-stress model (Monroe & Simons, 1991) and one part of the differential susceptibility model (Belsky & Pluess, 2009) posit that children carrying unfavorable intrapersonal characteristics, such as high (versus low) negative emotionality (Slagt et al., 2016), tend to be more vulnerable to the negative influences of adverse environments (e.g., deprivation). Supporting this proposition, as compared to children with low negative emotionality, children with high negative emotionality, when exposed to high family socioeconomic risks or limited maternal stimulation (both are proxies for early deprivation), were more likely to demonstrate lower levels of academic readiness and achievement in reading and math subjects (e.g., Collet et al., 2024; Wang et al., 2017). Similarly, in a predominantly low-income, population-based longitudinal sample of 1,259 children followed from birth (Raver et al., 2013), chronic exposure to high financial strains (across 15 to 35 months) was found to be associated with lower executive functioning performance at 48 months for children with temperamental profiles of high reactivity, but not for those who were less temperamentally reactive.

Other patterns have also been identified for the interaction between child negative emotionality and adverse environments in shaping cognitive development. As compared to those with low negative emotionality, results of a few studies indicated that children with high negative emotionality were more likely to exhibit less impaired or even enhanced cognitive functioning (e.g., reward-oriented problem-solving or spatial cognitive ability) when exposed to adverse living environments characterized by limited earned income and maternal disengagement (e.g., Suor et al., 2017) or authoritarian parenting (Cha, 2018). Relatedly, Suor and colleagues (2017) also identified that, in the face of adverse environments, children with a “hawk” temperament (Korte et al., 2005) tended to expend more energy in securing valuable resources. These children also displayed bolder, more proactive coping strategies to address their emotional, material, or cognitive needs for survival and flourishing (Suor et al., 2017), indicating their adaptive agency and initiatives in stressful living situations. Essentially, the “hawk” temperament, first introduced by Korte et al. (2005), is characterized by a constellation of interrelated dispositional traits, including heightened levels of aggressiveness, boldness, activity, and approach. It is featured functionally by bold, quick, and direct strategies for accessing resources and defeating threats. Conceptually, children carrying the “hawk” temperament are deemed to share some core characteristics – dominant negative affect (e.g., anger, frustration) and high activity levels – with children with high negative emotionality (Hentges et al., 2023; Sturge-Apple et al., 2012; Suor et al., 2017). As such, children with high negative emotionality or the other “hawk” temperament characteristics may develop certain domain-specific cognitive abilities in an “environment-fitting” manner, ultimately increasing their chances of survival and success (Frankenhuis & de Weerth, 2013; Frankenhuis et al., 2016). However, despite these extant studies, there is still a paucity of research that systematically examines how child negative emotionality may interact with early environmental adversities (e.g., deprivation) to shape *various components of* cognitive ability. Addressing this gap may yield a more nuanced understanding of how and why children exposed to early deprivation-related adversities may display various patterns of cognitive development.

### The present study

Against the aforementioned backdrop, we sought to test a developmental cascade model in which exposure to deprivation in early childhood was linked to academic performance in middle adolescence through cognitive ability in middle childhood. In doing so, we particularly aimed to illuminate the potential nuances implicated in this cascade by (a) breaking down the broad cognitive ability into specific sub-components and (b) testing child negative emotionality as a potential vulnerability or plasticity factor in shaping the consequences of early deprivation for subsequent cognitive outcomes. Based on prior research, we offered the following hypotheses.

First, early deprivation would be negatively and indirectly associated with academic performance in middle adolescence via a negative association with the *broad* cognitive ability in middle childhood (Hypothesis I). Second, child negative emotionality would moderate the link between early deprivation and later broad cognitive ability (Hypothesis II). Notably, prior research indicates complexity in the potential patterns for this moderation. In line with the diathesis-stress model (Monroe & Simons, 1991) and one part of the differential susceptibility model (Belsky & Pluess, 2009), child negative emotionality could be a vulnerability factor that likely amplify the negative consequences of early adversities, such that the negative link between early deprivation with later broad cognitive ability would be more pronounced for children with higher (versus lower) negative emotionality. Yet, it is also possible that high negative emotionality could be protective in the face of early deprivation exposure, given that the “hawk-like” characteristics may confer children “adaptive strengths” to cope with stressful living situations (Frankenhuis & de Weerth, 2013; Frankenhuis et al., 2016). That is, the negative link between early deprivation and later broad cognitive ability would be less pronounced for children with higher (versus lower) negative emotionality. Taken altogether, we acknowledged such complexity and did not offer a definite hypothesis for the specific interactive patterns in regard to this moderation. Third, regardless of the interaction patterns identified for Hypothesis II, we expected a conditional indirect effect would also be further identified, in which the association of early deprivation with academic performance in middle adolescence via the broad cognitive ability in middle childhood would be shaped by child negative emotionality, due to its moderating role in the link between deprivation and later cognitive ability (Hypothesis III).

Further, given the multifaceted nature of cognitive ability and the research need for greater nuance and specificity, we expected that the potential moderating effects of child negative emotionality for the cognitive consequences of early deprivation would vary as a function of the cognitive ability domain/component (Hypothesis IV). Specifically, four cognitive ability sub-components available in the used dataset were considered in the present study, including working memory, language ability, reading comprehension, and problem-solving ability domains. Notably, in all analyses, we controlled for the effect of early life threat and unpredictability to highlight the unique influences of early deprivation (McLaughlin et al., 2021; Phillips et al., 2023; Usacheva et al., 2022). In addition, a series of demographics were also considered, including the child’s gender and age, parents’ age and educational level, and the family economic status, which has been associated with adolescent academic outcomes in prior research (Henry et al., 2020; Liu J. et al., 2020).

## Method

### Participants and procedures

Participants were drawn from the Future of Families and Child Wellbeing Study (FFCWS). This ongoing longitudinal study follows 4,898 American children born between 1998 and 2000, located in 20 different cities, covering mid-sized cities, metropolises, and large urban areas (James et al., 2021; Reichman et al., 2001). Notably, at the initial sampling, approximately three-quarters of the infants had unmarried parents at birth. The FFCWS consists of three sub-studies: the Primary Caregiver Study (PCG, Core Study), which includes surveys of mothers, fathers, and/or other caregivers; the In-Home Study, which covers surveys of primary caregivers by the main interviewer, standardized tests conducted on children, and observations of the target family’s structure, parenting behaviors, and parent–child relationships; and the Child Care Centers and Teachers’ Study, which gathers information from childcare providers and teachers of the participating families’ children. Here we used the first two sub-studies. More details about FFCWS can be obtained at: https://ffcws.princeton.edu.

Out of the seven waves of data (from birth to age 22), the present study selected the second (at age 1, Y1; negative emotionality), third (at age 3, Y3; early life adversities), fifth (at age 9, Y9; cognitive ability), and sixth (at age 15, Y15; academic performance) waves of data for analysis. Participants were included in the analytic sample if they provided valid data on the early adversities dimension at Y3. The final sample consisted of 2,085 children (*M*_age_ = 3.19, *SD* = 0.24) and their caregivers at Y3 (survey time: 2001–2003). Among them, 1,795 families were followed six years later at Y9 (survey time: 2007–2010; retention rate: 86.09%); and 1,806 families participated in the assessments at Y15 (survey time: 2014–2017; retention rate: 86.62%). Nearly half of the children (47.8%) were girls. For mothers’ race/ethnicity, 18.7% identified as White, 54.8% Black, 23.1% Hispanic, and 3.4% Other. Regarding family economic status, 46.5%, 24.7%, 12.2%, and 12.2% of the families scored below 100%, between 100 and 200%, between 200 and 300%, and above 300% on the income-to-needs ratio, respectively. The proportion of fathers and mothers with “high school education or less” was 62.6% and 58.0%, respectively. The *mean* age of fathers and mothers was 30.55 ± 7.25 and 27.82 ± 5.89, respectively.

The Multivariate Analysis of Variance (MANOVA) was performed to detect potential differences in key deprivation variables at Y3 between adolescents who participated in all three waves of assessments (*n* = 1,700) and those who did not (i.e., tests of attrition bias). The MANOVA omnibus test showed a significant result with *F* (3, 2081) = 4.995, *p* = .002. Post hoc tests revealed that adolescents who participated in all three waves experienced less cognitive deprivation [*t* (539.658) = 3.188, *p* = .002] and less emotional deprivation [*t* (546.480) = 2.346, *p* = .019] compared to those who did not. However, the effect sizes for these differences were quite small (effect size of partial η^2^ = .005 and .003), which were well below the criterion value of partial η^2^ > .14 for a non-negligible difference (Bandalos, 2002; Cohen, 1988). Thus, attrition bias appeared to be negligible.

### Measures

#### Academic performance (Y15)

At Y15, adolescents reported their most recent grades for each of the four core subjects (language arts, math, social science, and science) on a four-letter scale (A, B, C, and D or lower). These letter grades were coded into corresponding numerical values (*A* = 4, *B* = 3, *C* = 2, D or lower = 1) for ease of subsequent modeling analyses and result interpretation, which is in line with the approach used by Gaydosh and McLanahan (2021). Then, a latent construct was created using the four grades as four manifest indicators. In this study, McDonald’s omega coefficient for reliability (as suggested by Hayes & Coutts, 2020; hereinafter the same) was 0.690.

#### Deprivation (Y3)

In line with previous research (Kasparek et al., 2023), three types of early deprivation were considered, including cognitive, emotional, and material components. Cognitive deprivation was assessed using the Home Observation for Measurement of the Environment Scale (HOME; Bradley & Caldwell, 1988). Primary caregivers rated the number of different types of toys and books available to the child at home on a four-point scale from 1 (*none*) to 4 (*five or more*). This study selected eight items reflecting the richness of cognitive stimuli available to the child at home, such as “*How many toys that make music does the child have*?” and “*How many books does the child have*?” These items were reverse-scored, and an average score was calculated. Higher scores indicate greater cognitive deprivation. In this sample, McDonald’s omega coefficient for reliability was 0.769.

Emotional deprivation was assessed during home visits. Using the HOME Scale (Bradley & Caldwell, 1988), researchers evaluated parent–child interactions in the home on a binary scale (1 = *yes* or 0 = *no*). This study selected three items based on the definition of emotional deprivation: *During the visit, the parent spontaneously praised the child at least twice; the parent’s voice conveyed positive feelings toward the child; and the parent hugged or kissed the child at least once*. These items were reverse scored, and a total score was calculated. Higher scores indicate greater emotional deprivation. In this sample, McDonald’s omega coefficient for reliability was 0.700.

Material deprivation was assessed using the U.S. Department of Agriculture’s Food Security Survey Module (Bickel et al., 2000) completed by the primary caregiver. This questionnaire contains 18 items to assess the household’s food availability and access over the past 12 months, such as “*Did you worry that food would run out before you got more money*?” and “*Did you skip meals or cut meal size to make food last longer*?” The primary caregiver responded with “*yes*” (1) or “*no*” (0) to these questions. Following the methodology of Hatem et al. (2020), all 18 responses were summed, and households were classified based on whether the number of affirmative responses was three or more (up to 18). This criterion was used as the measure of early material deprivation.

#### Cognitive ability (Y9)

This study measures children’s cognitive ability at age 9 using four standardized psychological tests (i.e., Broad Cognitive Ability; Grotzinger et al., 2019). These tests include the Wechsler Intelligence Scale for Children (WISC-IV; Wechsler, 2003) for working memory, the Peabody Picture Vocabulary Test (PPVT-III; Dunn & Dunn, 1997) for language ability, and the Woodcock-Johnson Tests of Achievement (Passage Comprehension and Applied Problems; Woodcock et al., 2001) for reading comprehension and problem-solving abilities. Standard scores (which reflect the performance of the focal child relative to their same-aged peers) for these four tests were used as manifest indicators for a broader latent construct of cognitive ability, with higher scores indicating higher levels of overall cognitive ability. To validate the construct validity of cognitive ability as a latent construct here, we conducted a first-order confirmatory factor analysis (CFA). The results indicated good construct validity (χ^2^ = 25.849, *df* = 2, *p* < .001, RMSEA = .082 with 90% CI [.056, .111], CFI = .990, SRMR = .017), with standardized factor loadings ranging from .552 to .843 and all relevant *ps* < .001. Further detailed information about these standard scores can be found in the relevant section on the FFCWS website: https://ffcws.princeton.edu/.

#### Negative emotionality (Y1)

Consistent with previous research (Golm & Brandt, 2024), this study adopted three items from the Emotionality, Activity, and Sociability Temperament Questionnaire developed by Mathiesen & Tambs (1999) to assess child negative emotionality at age 1 (i.e., “*Child often fusses and cries,*” “*Child gets upset easily,*” and “*Child reacts strongly when upset*”). Mothers rated how well these items described their child on a five-point scale from 1 (*not characteristic or typical of your child*) to 5 (*very characteristic or typical of your child*). Then, a latent construct was created using the three negative emotionality item scores as indicators. In this sample, McDonald’s omega coefficient for reliability was 0.626. To validate the construct validity of negative emotionality as a latent construct, we conducted a first-order CFA. In this 3-indicator saturated model, standardized factor loadings ranged from .466 to .771, with all relevant *ps* < .001.

Notably, negative emotionality at Y1 was used in the present study due to the following considerations. First, one of our central research questions was to test how child temperamental characteristics might interact with early deprivation exposure to predict later cognitive and academic outcomes. Child temperament was only measured at Y1 in the FFCWS dataset. We had to work within the constraints of the existing data. Second, there is a wealth of literature supporting the substantial mean-level and rank-order stability and continuity of child negative emotionality over time (Shiner, 2015) from infancy/early childhood through later developmental periods (e.g., Belsky et al., 1991; Carranza Carnicero et al., 2000; Casalin et al., 2012; Durbin et al., 2007; Kopala-Sibley et al., 2018; Putnam et al., 2001). Thus, negative emotionality measured at Y1 might be a somewhat reliable proxy for negative emotionality at subsequent time points. Nevertheless, we acknowledge the use of negative emotionality assessed at Y1 as a limitation of the present analyses (see the Limitations and Future Directions section for details).

#### Control variables (Y3)

Given the potential impacts of demographic factors on child’s academic performance (Henry et al., 2020; Liu J. et al., 2020), we considered a series of demographic variables as control variables in analyses, including child gender (1 = *boys*; 2 = *girls*), child age in years, bi-parental age in years, bi-parental educational level (1 = *less high school*; 2 = *high school or equivalent*; 3 = *technical college*; 4 = *university graduation or above*), and the family socioeconomic status (percentage on the income-to-needs ratio; 1 = 0–49%; 2 = 50–99%; 3 = 100–199%; 4 = 200–299%; 5 = 300% and above). Notably, to demonstrate the unique effect of deprivation, we also included early life threat and unpredictability as control variables. Referencing the methodology of Miller et al. (2021), the *threat* dimension of early life adversity was assessed at Y3 using two subscales from the Parent–Child Conflict Tactics Scales (CTSPC; Straus et al., 1998): Emotional Abuse and Physical Violence. The Emotional Abuse subscale includes 5 items, such as “*the number of times the primary caregiver swore at the child in the past year*”; and the Physical Violence subscale also consists of 5 items, such as “*the number of times the primary caregiver spanked the child’s bottom with a belt or hard object in the past year.*” The primary caregiver responded to these items on a seven-point scale from 0 (*never happened*) to 6 (*more than 20 times*). We used the average score of these 10 items, with higher scores indicating more threatening exposures. Here, McDonald’s omega coefficient was 0.795. Referencing previous research methodologies (Hoffman et al., 2024; Williams, 2023), the *unpredictability* dimension of early life adversity was measured with five items. These items covered changes in the family environment from age 1 to 3, including changes in the father’s employment, changes in parents’ marital status, changes in co-resident partners with the mother, and the number of relocations by both the father and the mother. We used binary coding (0 = *consistent*, 1 = *inconsistent*) to describe these family environment unpredictable characteristics between children ages 1 and 3. Sum scores of the 5 items were used, with higher scores indicating higher levels of early life unpredictability.

### Analytic strategies

This study used SPSS 29.0 and M*plus* 8.7 (Muthén & Muthén, 1998–2017) for data processing and analysis. The missing values were primarily due to participant attrition across waves (*n* = 303, see Table S1 for more details per variable). The current study investigated whether the data were missing at random using Little’s Missing Completely at Random (MCAR) test. Results showed it was identified as Missing at Random (MAR; see Supplementary Materials for more details about the justification for the Full Information Maximum Likelihood (FIML) treatment of missing data). Considering that FIML can maximize the use of available data and performs better in handling missing data in longitudinal studies as compared to direct data deletion or other conventional methods (Raykov, 2005), this study opted for FIML to address missing data.

To test Hypothesis I, we linked the latent variable of early “*deprivation*” at age 3, which was indicated by three forms of deprivation, with the latent variable of academic achievement at age 15, which was indicated by grade levels in four subjects. Further, the latent variable of cognitive ability at age 9, which was indicated by child performance in four cognitive tests, was included as a potential mediator (Model I). Several model fit indices were utilized to assess the model adequacy (Kline, 2015), including a non-significant chi-square statistic (χ^2^), Comparative Fit Index (CFI) > .90, Root Mean Square Error of Approximation (RMSEA) < .08, and Standardized Root Mean Square Residual (SRMR) < .08. Notably, with large sample sizes, a significant chi-square statistic (χ^2^) could also be acceptable (Byrne, 2013). The bias-corrected non-parametric percentile bootstrapping technique with 5000 resamples was employed to test for indirect effects and estimate confidence intervals (Preacher & Hayes, 2008). If the 95% confidence interval surrounding an unstandardized indirect effect does not include zero, this indirect effect would be considered significant.

Then, we used the latent moderated structural equation (LMS, Maslowsky et al., 2015) models to test our Hypothesis II. LMS is advantageous over the more conventional moderator analyses as the yielded estimates of interactions are less impacted by measurement errors (Maslowsky et al., 2015). First, as recommended by Maslowsky et al. (2015), prior to estimating structural models, we first did a measurement model to assess whether the four latent variables fit the data adequately, including deprivation at age 3, cognitive ability at age 9, academic performance at age 15, and negative emotionality at age 1 (indicated by three kinds of behaviors showing negative emotionality). Second, an LMS model (Model II) was run to determine whether negative emotionality was a moderator of the association between early deprivation adversity and later cognitive ability/academic performance. As conventional model fit indices are not provided for LMS models, a two-step procedure for latent moderator analyses was used (Maslowsky et al., 2015). In the first step, the measurement model without the latent interaction term was examined to determine model fit (called Model XX-0, where XX stands for the specific model number, ranging from II to VI, the same as below). For the second step, the model with the latent interaction term was added (named Model XX-1). Then, to evaluate whether adding the latent interaction term (early deprivation × child negative emotionality) would result in a significant improvement in the model fit, the log-likelihood ratio test was used to compare Model XX-1 with Model XX-0 (Maslowsky et al., 2015), using the following equation: *D* = −2[(log-likelihood for Model XX-0) – (log-likelihood for Model XX-1)]. The values of *D* are approximately distributed as χ^2^ with degrees of freedom (*df*), which is calculated by subtracting the number of free parameters in Model XX-0 from the number of free parameters in Model XX-1 (Maslowsky et al., 2015).

After identifying a significant interaction (if any), to reveal the specific interaction patterns, we used the Johnson–Neyman technique in M*plus* to identify the full range of moderator values where the simple slopes were significant (i.e., the region of significance test; Lin, 2020). Then, among those levels of the moderator (i.e., negative emotionality) for which the simple slopes were significant, both higher and lower levels (e.g., *Mean* + 1*SD* and *Mean* − 1*SD*, respectively; Aiken & West, 1991) of the moderator were used to illustrate the patterns for the identified interactive effects. Last, to test the conditional indirect effects (Hypothesis III), we used the “Model Constraint” command in M*plus* to define the indirect effects at the higher and lower levels of child negative emotionality and tested the indirect effects using the bootstrapping technique with 5,000 resamples.

Finally, to test Hypothesis IV, we further broke down the broad construct of cognitive ability into its four sub-components to identify which specific aspects of cognitive ability were influenced by early deprivation exposure and its interaction with child negative emotionality (i.e., Model III to Model VI). A series of control variables, including early life threat and unpredictability, were controlled for in all the models by specifying them as predictors for academic performance.

## Results

**Figure 1.**
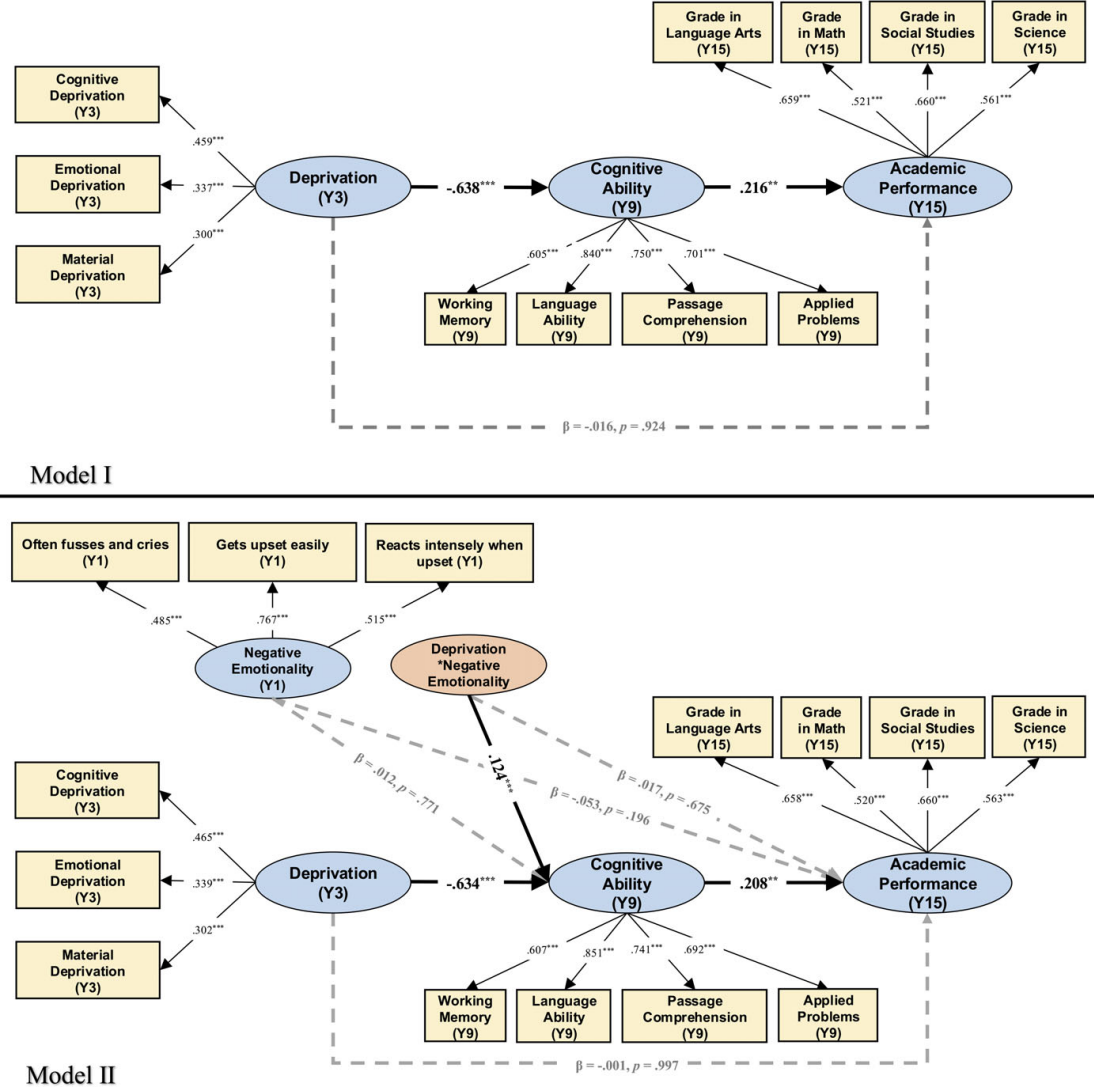
Results of Model I and Model II. *Notes.* Y1, one-year-old; Y3, three-year-old; Y9, nine-year-old; Y15, fifteen-year-old. Black, solid lines indicate paths with significant coefficients, whereas grey, dashed lines indicate paths with non-significant coefficients. standardized coefficients are reported. *p < .05; **p < .01; ***p < .001 (two-tailed). The predicting paths and correlation lines involving covariates are omitted for clarity purposes. Covariates include child gender, child age, parents’ age, parents’ education level, and family socioeconomic status. Threat and unpredictability dimensions of early life adversity were also included as covariates, to demonstrate the unique effect of early deprivation.

Descriptive statistics and bivariate correlations among key study variables and between key study variables and covariates are presented in Table 1. The model I as depicted in Figure 1 fit the data well: χ^2^ (111) = 351.674 with *p* < .001, RMSEA = .032 with 90% CI [.028, .036], CFI = .950, and SRMR = .026. After controlling for the covariates at Y3, exposure to deprivation at Y3 was negatively associated with cognitive ability at Y9 (β = −.638, *p* < .001), which, in turn, was positively related to academic performance at Y15 (β = .216, *p* = .004). Results of bootstrapping analyses indicated that the indirect effect from early deprivation to academic performance through cognitive ability was significant: *B* = −.322, *S.E.* = .303, 95% CI [−.799, −.047], β = −.138. However, exposure to deprivation at Y3 was not directly associated with academic performance at Y15 (β = −.016, *p* = .924).

**Table 1.** Descriptive statistics for and bivariate intercorrelations among key study variables and between key study variables and covariates

Variables	01	02	03	04	05	06	07	08	09	10	11	12	13	14	*M*	*SD*	
**Key Study Variables**																	
01. Cognitive Deprivation (Y3)	--																
02. Emotional Deprivation (Y3)	**.187**	--															
03. Material Deprivation (Y3)	**.102**	**.098**	--														
04. Working Memory (Y9)	**−.144**	**−.080**	**−.096**	--													
05. Language Ability (Y9)	**−.287**	**−.201**	**−.143**	**.332**	--												
06. Passage Comprehension (Y9)	**−.213**	**−.187**	**−.140**	**.470**	**.603**	--											
07. Applied Problems (Y9)	**−.201**	**−.144**	**−.122**	**.457**	**.549**	**.648**	--										
08. Grade in Language Arts (Y15)	**−.106**	−.022	**−.072**	**.066**	**.172**	**.125**	**.138**	--									
09. Grade in Math (Y15)	−.040	−.036	.000	**.088**	**.122**	**.104**	**.185**	**.382**	--								
10. Grade in Social Studies (Y15)	**−.149**	**−.093**	**−.080**	**.145**	**.233**	**.231**	**.244**	**.416**	**.304**	--							
11. Grade in Science (Y15)	**−.094**	**−.068**	−.027	**.084**	**.140**	**.139**	**.127**	**.363**	**.295**	**.384**	--						
12. Negative Emotionality #1 (Y1)	**.158**	**.079**	**.072**	**−.068**	**−.154**	**−.118**	**−.104**	**−.071**	−.040	**−.117**	**−.106**	--					
13. Negative Emotionality #2 (Y1)	**.100**	**.074**	**.090**	**−.050**	**−.125**	**−.083**	**−.069**	**−.050**	−.016	**−.075**	**−.097**	**.360**	--				
14. Negative Emotionality #3 (Y1)	−.004	.037	.031	−.030	−.035	.001	−.023	−.009	−.010	−.037	**−.050**	**.247**	**.408**	--			
**Covariates**																	
Child gender	.016	−.040	−.041	**.084**	.015	**.141**	**.060**	**.088**	**.072**	**.082**	**.074**	−.025	−.028	−.025	47.8 ^a^		
Child age in years	**.071**	−.023	−.007	**−.076**	**−.091**	**−.079**	**−.059**	.021	.045	.049	.015	.041	.035	−.017	3.186	.237	
Maternal age in years	**−.114**	**−.124**	**−.093**	**.054**	**.183**	**.100**	**.132**	**.095**	**.059**	**.119**	**.084**	**−.066**	**−.095**	−.016	27.820	5.894	
Paternal age in years	**−.093**	**−.107**	−.045	.041	**.197**	**.089**	**.099**	**.071**	.053	**.125**	.040	**−.055**	**−.054**	.022	30.550	7.251	
Maternal educational level	**−.285**	**−.179**	**−.159**	**.151**	**.364**	**.265**	**.234**	**.177**	**.144**	**.213**	**.146**	**−.128**	**−.158**	.025	2.240	.985	
Paternal educational level	**−.290**	**−.162**	**−.149**	**.166**	**.346**	**.263**	**.242**	**.200**	**.149**	**.177**	**.076**	**−.111**	**−.106**	−.021	2.160	.977	
Family socioeconomic status	**−.335**	**−.217**	**−.226**	**.146**	**.390**	**.295**	**.297**	**.159**	**.126**	**.218**	**.109**	**−.119**	**−.127**	−.036	2.740	1.392	
																
*Means*	1.860	0.458	0.186	9.290	92.080	92.210	97.470	2.892	2.716	2.902	2.834	2.463	2.742	3.347			
*Standard Deviations*	0.540	0.824	0.389	2.771	14.527	14.279	16.102	0.879	0.946	0.929	0.923	1.369	1.493	1.481			

*Note.* Sample sizes ranged from 1,200 to 2,085, representing the number of participants for each pair of correlations, given missing data for some variables. Bolded coefficients were significant with at least *p* < .05 (two-tailed). Y1, one-year-old; Y3, three-year-old; Y9, nine-year-old; Y15, fifteen-year-old. Negative Emotionality #1: *The child often fusses and cries*. Negative Emotionality #2: *The child gets upset easily*. Negative Emotionality #3: *The child reacts strongly when upset*. ^a^ The percentage of girls.

Before conducting latent moderated structural equation analyses, we assessed the measurement model comprising all 4 latent variables of deprivation (Y3), negative emotionality (Y1), cognitive ability (Y9), and academic performance (Y15). Correlations among latent variables were all significant (|*r*| ranged from .142 to .635, see Table S2 for details). Model fit was good for this measurement model: χ^2^ (69) = 193.325 with *p* < .001, RMSEA = .029 with 90% CI [.025, .034], CFI = .973, and SRMR = .031. After adding the structural model (see Table S3 for details), the Model II-0 demonstrated adequate fit, χ^2^ (159) = 478.573 with *p* < .001, RMSEA = .031 with 90% CI [.028, .034], CFI = .944, and SRMR = .029. The loglikelihood comparison test between Model II-0 and Model II-1 (i.e., with latent variable interaction, Model II as depicted in Figure 1) indicated that adding the latent interaction effect significantly improved model fit, χ^2^(2) = 13.600, *p* = .001. Besides, a decrease in AIC also indicated the improved model fit of the moderated mediation model (ΔAIC = −9.600). The latent deprivation (Y3) × negative emotionality (Y1) interaction was significantly positively associated with cognitive ability at Y9 (β = .124, *p* < .001).

**Figure 2.**
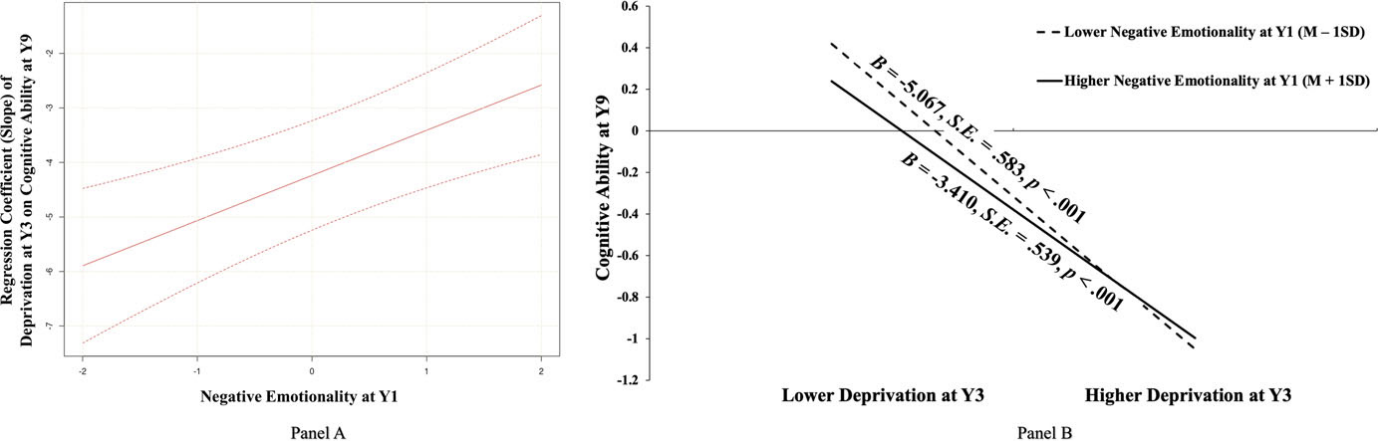
The moderating role of negative emotionality at Y1 in the association between early deprivation at Y3 and cognitive ability at Y9. *Notes.* **Panel (A):** An illustration of the slope of early deprivation at Y3 on cognitive ability at Y9 at different child negative emotionality levels at Y1 using the johnson–Neyman technique. The vertical axis represents the unstandardized value of the slope. Negative emotionality is a normally distributed latent variable with a mean of 0 and a variance of 1. The upper and lower dashed lines represent the 95% confidence intervals for the slope estimate. **Panel (B):** A pattern illustration of the identified interaction between early deprivation and child negative emotionality in predicting later cognitive ability at Y9. Y1, one-year-old; Y3, three-year-old; Y9, nine-year-old. *B*, *S.E.*, and *p* represented unstandardized coefficients, corresponding standard errors, and the significance level, respectively.

Then, using the Johnson–Neyman Technique in M*plus*, we plotted the slope of deprivation at Y3 on cognitive ability at Y9 as a function of the negative emotionality (Y1) values ranging from *Mean* – 2 *SD* to *Mean* – 2 *SD* (covering 95.45% value range due to the normal distribution assumption about the latent variable). The results (as depicted in Figure 2, Panel A) showed that deprivation at Y3 was always negatively associated with cognitive ability at Y9 whenever the negative emotionality (Y1) moderator was low (*Mean* – 2 *SD*) and high (*Mean* + 2 *SD*). To specifically describe the effect (slope) of deprivation at Y3 on cognitive ability at Y9 under high and low negative emotionality (Y1), and to compare the effect under the two conditions, we analyzed the simple slope and relative size when negative emotionality (Y1) took *Mean* – 1*SD* and *Mean* + 1*SD*. As further illustrated in Panel B of Figure 2, the negative association between deprivation at Y3 and cognitive ability at Y9 was significant for adolescents with both higher negative emotionality (Y1) (i.e., *Mean* + 1*SD*; *B* = −3.410, *S.E.* = .539, *p* < .001) and lower negative emotionality (Y1) (i.e., *Mean* – 1*SD*; *B* = −5.067, *S.E.* = .583, *p* < .001), but the strength of this association (e.g., absolute value of slope) was weaker for adolescents with higher (versus lower) negative emotionality (Y1) (*B* = 1.657, *S.E.* = .459, *p* < .001). Further, results of bootstrapping analyses for conditional indirect effects demonstrated that the indirect effects from early deprivation to academic performance through cognitive ability were significant among both adolescents with higher negative emotionality (Y1) (*B* = −.244, *S.E.* = .096, 95% CI [−.571, −.033]) and adolescents with lower negative emotionality (Y1) (*B* = −.363, *S.E.* = .136, 95% CI [−.785, −.044]), with the latter being significantly stronger than the former (*B* = .119, *S.E.* = .053, 95% CI [.018, .379]).

**Figure 3.**
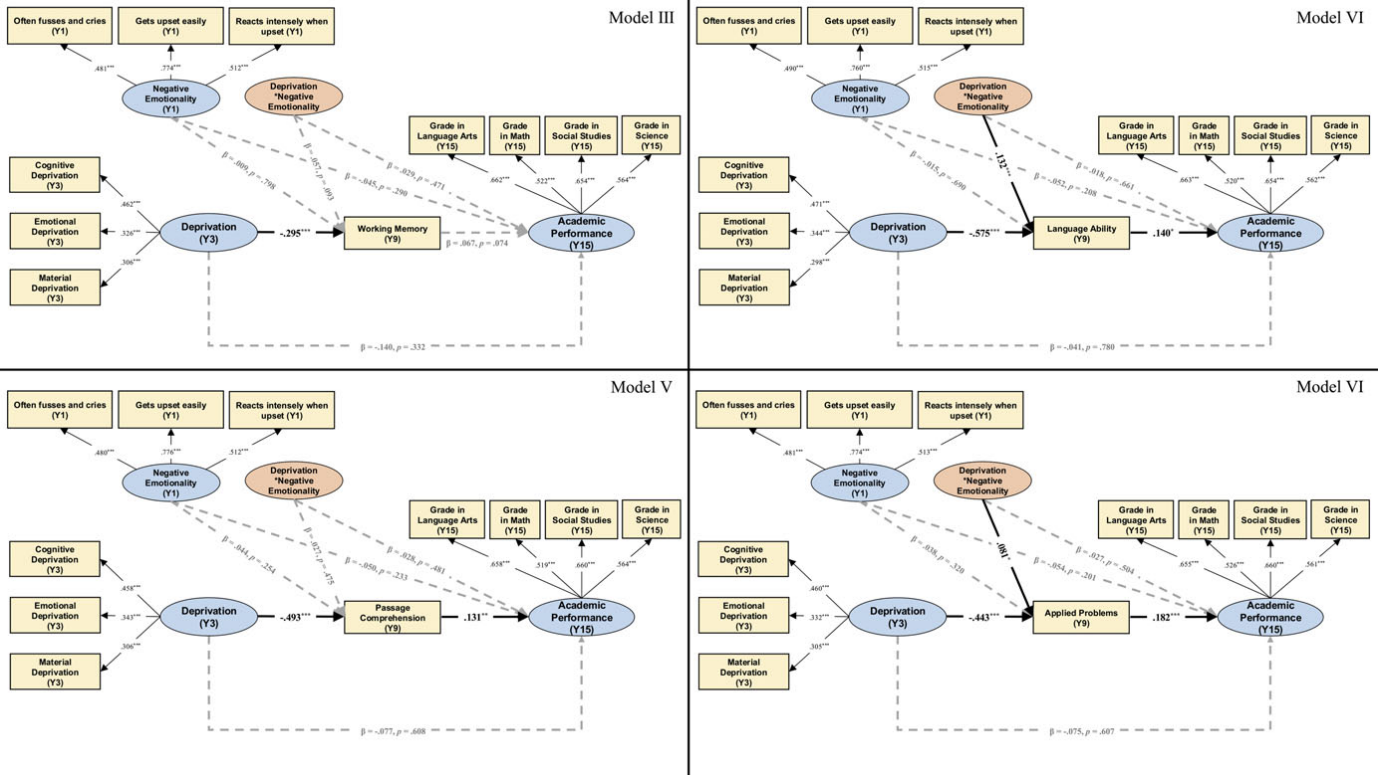
Results of Model III to Model VI with specific cognitive ability components (manifest variable) as the mediating mechanisms. *Notes.* Y1, one-year-old; Y3, three-year-old; Y9, nine-year-old; Y15, fifteen-year-old. Black, solid lines indicate paths with significant coefficients, whereas grey, dashed lines indicate paths with non-significant coefficients. standardized coefficients are reported. *p < .05; **p < .01; ***p < .001 (two-tailed). The predicting paths and correlation lines involving covariates are omitted for clarity purposes. Covariates include child gender, child age, parents’ age, parents’ education level, and family socioeconomic status. Threat and unpredictability dimensions of early life adversity were also included as covariates, to demonstrate the unique effect of early deprivation.

**Figure 4.**
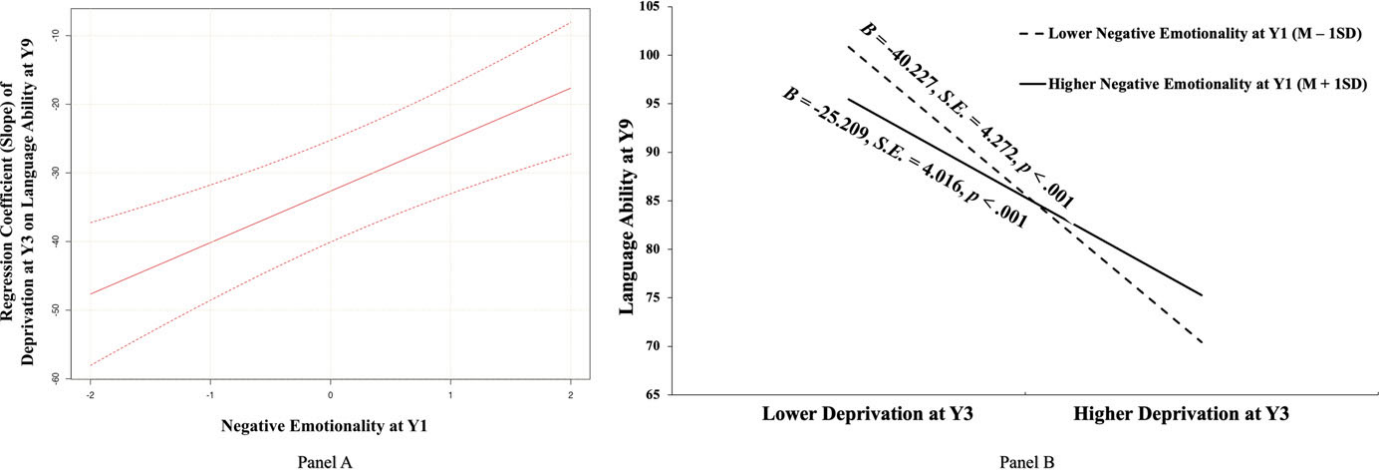
The moderating role of negative emotionality at Y1 in the association between early deprivation at Y3 and language ability at Y9. *Notes.* **Panel (A):** An illustration of the slope of early deprivation at Y3 on language ability at Y9 at different child negative emotionality levels at Y1 using the johnson–Neyman technique. The vertical axis represents the unstandardized value of the slope. Negative emotionality is a normally distributed latent variable with a mean of 0 and a variance of 1. The upper and lower dashed lines represent the 95% confidence intervals of the slope estimate. **Panel (B):** A pattern illustration of the identified interaction between early deprivation and child negative emotionality in predicting later language ability at Y9. Y1, one-year-old; Y3, three-year-old; Y9, nine-year-old. *B*, *S.E.*, and *p* represented unstandardized coefficients, corresponding standard errors, and the significance level, respectively.

**Figure 5.**
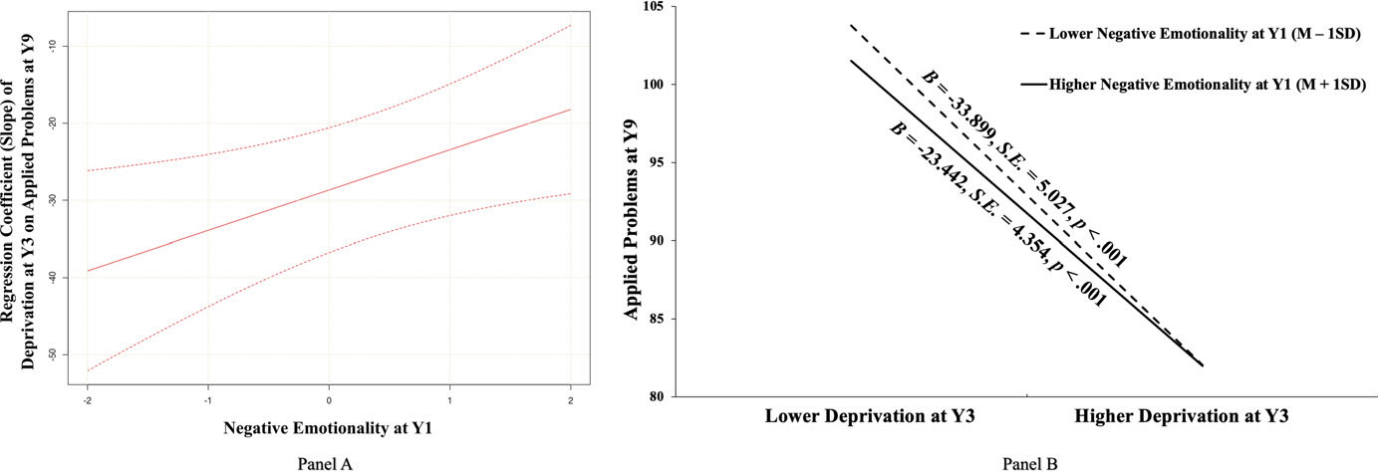
The moderating role of negative emotionality at Y1 in the association between early deprivation at Y3 and applied problems at Y9. *Notes.* **Panel (A):** An illustration of the slope of early deprivation at Y3 on applied problems at Y9 at different child negative emotionality levels at Y1 using the johnson–Neyman technique. The vertical axis represents the unstandardized value of the slope. Negative emotionality is a normally distributed latent variable with a mean of 0 and a variance of 1. The upper and lower dashed lines represent the 95% confidence intervals of the slope estimate. **Panel (B):** A pattern illustration of the identified interaction between early deprivation and child negative emotionality in predicting later applied problems at Y9. Y1, one-year-old; Y3, three-year-old; Y9, nine-year-old. *B*, *S.E.*, and *p* represented unstandardized coefficients, corresponding standard errors, and the significance level, respectively.

To further clarify which specific aspects of cognitive ability served as the process mechanisms, we broke down the broad construct of cognitive ability into four sub-components and examined the potential mediating roles of working memory, language ability, reading comprehension, and problem-solving in such associations separately (Figure 3). Notably, in all four separate models, exposure to deprivation at Y3 was not directly associated with academic performance at Y15 (βs = −.140 ∼ −.041, *ps* = .332 ∼ .780). According to bootstrapping statistics presented in Table 2, the overall indirect effect applied to three of the four components (except for working memory with a marginal significance), and the significant interaction terms emerged for language ability and applied problems. As presented in Figure 3, the latent deprivation (Y3) × negative emotionality (Y1) interaction was significantly and positively associated with language ability at Y9 (β = .132, *p* < .001) and applied problems at Y9 (β = .081, *p* = .019). Then, we plotted the slope of deprivation at Y3 on language ability and applied problems at Y9, respectively, as a function of the negative emotionality (Y1) values ranging from *Mean* – 2 *SD* to *Mean* + 2 *SD*. The results (as depicted in both Figures 4 and 5, Panel A) showed that deprivation at Y3 was always negatively associated with language ability and applied problems at Y9 whenever the negative emotionality (Y1) moderator was low (*Mean* – 2 *SD*) and high (*Mean* + 2 *SD*).

**Table 2.** The specific indirect effects in the four models (Model III to Model VI) with each of the four specific cognitive ability components (manifest variables) tested as the potential mediating mechanism separately

Specific Indirect Pathways Tested in the Models	Bootstrapped Estimates for Indirect Effects		
	*B*	*S.E.*	95% CI
Deprivation at Y3 → Working Memory at Y9 → Academic Performance at Y15	−.046	.044	[−.115, .017]
Deprivation at Y3 → Language Ability at Y9 → Academic Performance at Y15	−.184	.107	[−.422, −.025]
Deprivation at Y3 → Reading Comprehension at Y9 → Academic Performance at Y15	−.151	.095	[−.335, −.006]
Deprivation at Y3 → Applied Problems at Y9 → Academic Performance at Y15	−.187	.066	[−.323, −.083]

*Notes.* Y3, three-year-old; Y9, nine-year-old; Y15, fifteen-year-old. B, unstandardized coefficient; *S.E.*, standard error; and CI, the confidence interval for the unstandardized coefficient.

To specifically describe the effect (slope) of deprivation at Y3 on language ability and applied problems at Y9 under high and low negative emotionality (Y1), and to compare the effect under the two conditions, we analyzed the simple slope and relative size when negative emotionality (Y1) took *Mean* – 1*SD* and *Mean* + 1*SD*. As further illustrated in Panel B of Figure 4, the negative association between deprivation at Y3 and language ability at Y9 was significant for adolescents with both higher negative emotionality (Y1) (i.e., *Mean* + 1*SD*; *B* = −25.209, *S.E.* = 4.016, *p* < .001) and lower negative emotionality (Y1) (i.e., *Mean* – 1*SD*; *B* = −40.227, *S.E.* = 4.272, *p* < .001), but the strength of this association (e.g., absolute value of slope) was weaker for adolescents with higher (versus lower) negative emotionality (Y1) (*B* = 15.019, *S.E.* = 3.423, *p* < .001). Similarly, as illustrated in Panel B of Figure 5, the negative association between deprivation at Y3 and applied problems at Y9 was significant for adolescents with both higher negative emotionality (Y1) (i.e., *Mean* + 1*SD*; *B* = −23.442, *S.E.* = 4.354, *p* < .001) and lower negative emotionality (Y1) (i.e., *Mean* – 1*SD*; *B* = −33.899, *S.E.* = 5.027, *p* < .001), but the strength of this association (e.g., absolute value of slope) was significantly weaker for adolescents with higher (versus lower) negative emotionality (Y1) (*B* = 10.457, *S.E.* = 4.519, *p* = .021). Further, results of bootstrapping analyses demonstrated that the indirect effects from early deprivation to academic performance through language ability or applied problems were significant among adolescents with higher negative emotionality (Y1) (*B* = −.142, *S.E.* = .086, 95% CI [−.359, −.017] for language ability; *B* = −.153, *S.E.* = .057, 95% CI [−.268, −.067] for applied problems) and adolescents with lower negative emotionality (Y1) (*B* = −.227, *S.E.* = .130, 95% CI [−.512, −.031] for language ability; *B* = −.221, *S.E.* = .086, 95% CI [−.434, −.102] for applied problems), with the latter being significantly stronger than the former (*B* = .085, *S.E.* = .051, 95% CI [.016, .205] for language ability; *B* = .068, *S.E.* = .058, 95% CI [.004, .244] for applied problems).

## Discussion

Leveraging longitudinal data that spanned twelve years from early childhood through adolescence and utilized multiple informants and methods in assessments, this study sheds unique light on the complexity, nuance, and heterogeneity inherent in the cognitive consequences of early deprivation. Joining and extending prior work on the developmental consequences of poverty and other deprivation-related risk factors (e.g., Almas et al., 2016; Brooks-Gunn & Duncan, 1997; Liang et al., 2024; Reichman et al., 2001; Sameroff et al., 1987b; Sonuga-Barke et al., 2017; Wang et al., 2024), the present study makes a unique contribution by elucidating some explanatory processes (i.e., cognitive ability and its subcomponents) through which and by specifying some conditions (i.e., child negative emotionality) under which early deprivation exposure would be harmful for adolescent academic performance, while controlling for the effects of early life threat and unpredictability as informed by the Dimensional Model of Adversity (Ellis et al., 2022; McLaughlin et al., 2021; Usacheva et al., 2022).

### Developmental cascading chains involving cognitive ability and its subcomponents

Across all the tested models, no *direct* associations of early deprivation with academic performance during adolescence were identified. Instead, we consistently found the mediating roles of cognitive ability as a broad construct and its various subcomponents for the association between early deprivation and academic performance in adolescence. The detrimental implications of early deprivation for later cognitive development have been widely demonstrated in prior studies (e.g., Almas et al., 2016; Beckett et al., 2010; Miller et al., 2021; Vogel et al., 2021). Our study joins such studies and further highlights the unique effects of early deprivation by controlling for the effects of other key dimensions of early adversity (i.e., threat and unpredictability; Doom et al., 2023; McLaughlin et al., 2021; Usacheva et al., 2022). A recent meta-analysis indeed indicated that the association of deprivation with executive functioning was significantly larger than that for threat (Johnson et al., 2021). Threat may selectively interfere more with emotional responses and automatic regulation, while deprivation works more with higher-order cognitive functions (Miller et al., 2021; Phillips et al., 2023; Vogel et al., 2021). In addition, above and beyond threat, deprivation has been shown to be associated with poor language development and reading ability (e.g., Miller et al., 2021).

Similar to our identified indirect pathways, the results of a study by Sheridan and McLaughlin (2016) also indicated that deficits in cognitive control could be a bridging factor linking early deprivation experiences with subsequent school outcomes, such as attendance, graduation, and achievement. Uniquely, results of the present study demonstrate the indirect effects involving different cognitive ability components (i.e., language ability, reading comprehension, and applied problem-solving), suggesting that early deprivation exposure might comprehensively disrupt the normal development of various cognitive abilities and thus contribute to later academic difficulties. Built on the current study, future research will benefit from incorporating additional types of cognitive abilities and testing if their development is differentially vulnerable to early deprivation exposure (Young et al., 2024). Overall, leveraging longitudinal data that spanned 12 years from early childhood through adolescence, our study delineated a developmental cascade (Masten & Cicchetti, 2010) demonstrating that the accumulation of risks stemming from early deprivation exerts negative impacts on children’s development in the multiple cognitive domains, and as they progress in their development, the deficits in the cognitive domain likely further spread into the academic domain, leading to poor academic performance in the long run.

### The moderating role of child negative emotionality as an intrapersonal plasticity factor to fit the environment

Our findings also indicated that the negative association between early exposure to deprivation and later cognitive ability in middle childhood tended to be less pronounced among children with higher (versus lower) negative emotionality, which contributed to a weaker indirect effect on academic performance in middle adolescence among children with higher (versus lower) negative emotionality. Such findings are inconsistent with most previous studies that emphasize negative emotionality as a risk trait that confers heightened vulnerability in a “for worse” way in adverse environments (Belsky & Pluess, 2009; Frankenhuis & de Weerth, 2013; Monroe & Simons, 1991). From two samples – one from 436 families with twin siblings at their age 6 in Ohio, and the other from 1364 children and their families participated in the famous Early Child Care and Youth Development (SECCYD) project – Wang et al. (2017) both found that the negative associations between family socioeconomic status risks and children’s reading and math development were stronger among children with higher (versus lower) negative emotionality. Children with higher negative emotionality tend to experience higher levels of sadness, anger, and frustration in the face of stressful circumstances (Belsky et al., 1991; Slagt et al., 2016), which may account for their greater susceptibility to the detrimental influences of adverse environments.

However, another line of research and theories provides support to the patterns of interaction that we identified in the present study (Frankenhuis & de Weerth, 2013; Suor et al., 2017). As suggested by Suor et al. (2017), growing up in deprived environments, children with “hawk-like” temperamental traits (e.g., anger and frustration labeled dominant negative emotionality) may have the motivation to adopt a proactive behavioral strategy and develop certain cognitive skills at an accelerated pace to facilitate their effective coping with the demands of stressful living contexts (e.g., struggling with insufficient food and emotional neglect; Frankenhuis & de Weerth, 2013; Korte et al., 2005; Sturge-Apple et al., 2012). For example, consider a child with high negative emotionality and raised in a family characterized by poverty and parental neglect. This child’s intense reactions to family deprivation stressors may initially look like a hindrance. However, living in this deprived environment, this child’s heightened sensitivity and reactivity, conferred by negative emotionality, may enable quick detection of danger and also fuel actions to cope and/or escape, propelling or accelerating the development of language ability and problem-solving skills that are critical for survival under harsh conditions (Frankenhuis & Nettle, 2020). To sum up, in the face of deprivation, it is possible that children with high negative emotionality and/or other “hawk-like” temperamental characteristics may adapt themselves to mobilize their energy expenditure towards more proactive explorations and fuller uses of available (yet limited) resources, in order to recompense, at least to some extent, for their losses from families or caregivers and thus increase their survival chance (Suor et al., 2017). As a result, high negative emotionality and/or other “hawk-like” temperamental characteristics may attenuate the negative influences of early deprivation, including those related to the development of cognitive ability.

### Looking at the specific cognitive components: The salient role of language ability and applied problem-solving ability involved in the moderating associations

As to various sub-components for cognitive ability (i.e., working memory, language ability, reading comprehension, and applied problem-solving), we observed that both language ability and applied problem-solving ability played a role in the identified interaction between early deprivation and negative emotionality. Specifically, the negative link between early deprivation and subsequent language ability or applied problem-solving ability was *weaker* among children with *higher* (versus lower) negative emotionality.

When exposed to adverse conditions like early deprivation, children with high levels of negative emotionality may exhibit a higher dissatisfaction with their deprivation-focused environment, prompting them to proactively seek alternative cognitive, emotional, or material resources to fulfill their developmental and cognitive needs (Suor et al., 2017). Meanwhile, children may adaptively exhibit more advanced mind-reading abilities for survival purposes in a compensatory manner, as compared to children in non-threatening contexts (Frankenhuis & de Weerth, 2013). More receptive language abilities – comprehension of information presented through diverse means like auditory cues and language, physical actions and gestures, and visual signs and symbols – may serve as key fundamental skills in stressful circumstances for safety and survival (Lonigro et al., 2014). In addition, the salient role of language ability identified in the current analyses also aligns with prior research showing the severe language delays among children who had extremely profound deprivation exposure (e.g., children’s language outcomes in the Bucharest Early Intervention Project; Windsor et al., 2011, 2013).

The applied problem-solving test requires the child to analyze and solve math problems (Woodcock et al., 2001). To solve the problems, the child needs to listen to the problems, understand the procedures to follow, and then perform calculations. When doing so, the child also needs to decide not only on the appropriate mathematical operations to use but also on which numbers to include in the calculations. All these involved abilities are closely related to complex information processing, decision-making, and planning, which are also critical for safety and survival in risk contexts (Frankenhuis & de Weerth, 2013; Frankenhuis & Nettle, 2020; Frankenhuis et al., 2016). As such, for survival purposes in extreme environments, the more “hawk-like” temperamental characteristics may protect children’s such abilities from the deleterious influences of deprivation.

In addition, Young and colleagues (2024) recently found that socioeconomic adversity (as indicated by family income and neighborhood socioeconomic disadvantage) was linked to a decrease in overall cognitive performance, with the most notable reductions observed in Picture Vocabulary and Verbal Analogies subtests. In contrast to this general decline, however, performance on the Auditory Processing and Auditory–Visual Associations subtests appeared to be improved. Somewhat similar to our findings, these results also suggest that exposure to early adversities may shape later cognitive ability development in a more subtle and nuanced domain-specific pattern. Taking all these into consideration, more research is pressing and needed to explore such patterns more systematically.

### Limitations and future directions

Despite the substantial and abundant findings, several limitations should be noted. First, the data used are correlational, making any inferences about causality impossible. Second, the lack of baseline controls for both cognitive ability and academic performance (due to the unavailability of such data in the FFCWS project) diminished the credibility of the current findings.

Third, as child negative emotionality was only assessed at age 1 in the FFCWS project, we have to use it in our analyses to address research questions in regard to the interactions between temperamental characteristics and early adversities. Although negative emotionality tends to be relatively stable from infancy to later developmental periods (e.g., Carranza Carnicero et al., 2000; Casalin et al., 2012; Durbin et al., 2007; Kopala-Sibley et al., 2018; Putnam et al., 2001), it still remains open to environmental influences and may change over time to some extent (e.g., Perry et al., 2018; Zhou et al., 2023). Thus, future research can explore if the currently identified moderating effects would hold for negative emotionality measured either concurrently with early deprivation exposure or more proximally during adolescence. In addition to negative emotionality, future researchers may also test other seemingly more proximal, alternative moderators measured in adolescence that are available in the FFCWS dataset (e.g., adolescent psychopathology such as depression and anxiety, or household dysfunction; Andersen et al., 2021; Miller et al., 2021).

Fourth, the present study did not examine other important features of early deprivation (e.g., timing, chronicity, or variability) and how they may relate to later developmental performance (Jaffee & Maikovich-Fong, 2011; Walasek et al., 2024). For example, children experiencing parental neglect and other maltreatment across multiple developmental periods tend to have lower IQ scores than those exposed to such adversities in only one developmental period (Jaffee & Maikovich-Fong, 2011). Fifth, although four critical sub-components of cognitive ability (that are all available in the FFCWS project) were considered in the present analyses, we acknowledge that the structure and components of cognitive ability are complex. Future studies are needed to consider additional sub-components to adequately represent the construct of cognitive ability and test their roles in the current model.

Last, we also acknowledge that child negative emotionality could be a risk factor for more negative caregiver interactions (Golm & Brandt, 2024; Liu C. et al., 2020; van der Bruggen et al., 2010). That is, higher child negative emotionality may elicit subsequent higher levels of caregiver disengagement (i.e., deprivation) and/or higher levels of harsh parenting (i.e., threat), as caregivers may disengage due to a lack of parenting skills, caregiver distress, and/or low coping efficacy. When interpreting our results, this possibility should be cautiously considered. Yet, testing the child-driven effects in our models is beyond the scope of the present study, but it might constitute an interesting direction for future research.

## Conclusion

Our results demonstrated a developmental cascade spanning twelve years of life from early childhood through middle adolescence, in which cognitive ability in middle childhood accounted for how early life deprivation, as a distant and unique risk factor, would exert long-term adverse effects on academic performance in adolescence. Further, the cognitive ability of children with higher (versus lower) negative emotionality appeared to be less vulnerable to the adverse influences of early deprivation. More specifically, such cascading mechanisms conditioned by child negative emotionality applied particularly to language ability rather than the other components of cognitive ability. While preventing early deprivation is crucial, cognitive ability could be an important intervention target to reduce the negative long-term educational consequences for children who carry the burdens of early deprivation. Besides, child temperamental characteristics like negative emotionality should be considered when tailoring relevant interventions for cognitive development, especially for language and applied problem-solving ability.

## Supporting information

10.1017/S0954579425100813.sm001 Wang et al. supplementary material Wang et al. supplementary material

## Data Availability

Analytical data for this study are not available because the original data are copyrighted to the Future of Families and Child Wellbeing Study. Anyone who is interested in the publicly available data of FFCWS is suggested to apply directly through this website: https://ffcws.princeton.edu.

## Supplementary material

The supplementary material for this article can be found at https://doi.org/10.1017/S0954579425100813.

## Pre-registration statement

The study’s design and analytic plan were not pre-registered. All analysis codes and materials are available upon request from the corresponding author
