# Supplementary material for: Linking deprivation in early childhood with academic performance in middle adolescence through cognitive ability in middle childhood: Nuance by specific cognitive component and heterogeneity by child negative emotionality
Source: Dev Psychopathol. 2025 Oct 24:1–17. Online ahead of print. doi: 10.1017/S0954579425100813 (PMC12825954; doi:10.1017/S0954579425100813)
Supplement: Wang et al. supplementary material [file S0954579425100813sup001.docx]

Supplementary Materials for

**Linking Deprivation in Early Childhood with Academic Performance in Middle Adolescence Through Cognitive Ability in Middle Childhood: Nuance by Specific Cognitive Component and Heterogeneity by Child Negative Emotionality**

**Justification for the Treatment of Missing Data**

Recommended by Schlomer et al. (2010), we first reported the amount of missing data across key study variables below.

**Table S1.** The amount of missing data across the variables of interest in this study.

| Key Study Variables | Valid number | Missing number | Rate of missingness |
| --- | --- | --- | --- |
| 01. Cognitive Deprivation (Y3) | 2085 | 0 | 0 |
| 02. Emotional Deprivation (Y3) | 2085 | 0 | 0 |
| 03. Material Deprivation (Y3) | 2085 | 0 | 0 |
| 04. Working Memory (Y9) | 1782 | 303 | 14.53 |
| 05. Language Ability (Y9) | 1773 | 312 | 14.96 |
| 06. Passage Comprehension (Y9) | 1763 | 322 | 15.44 |
| 07. Applied Problems (Y9) | 1769 | 316 | 15.16 |
| 08. Grade in Language Arts (Y15) | 1644 | 441 | 20.19 |
| 09. Grade in Math (Y15) | 1648 | 437 | 20.96 |
| 10. Grade in Social Studies (Y15) | 1535 | 550 | 26.38 |
| 11. Grade in Science (Y15) | 1616 | 469 | 22.49 |
| 12. Negative Emotionality-Item 1 (Y1) | 1998 | 87 | 4.17 |
| 13. Negative Emotionality-Item 2 (Y1) | 1999 | 86 | 4.12 |
| 14. Negative Emotionality-Item 3 (Y1) | 1996 | 89 | 4.27 |

***Note.*** Y1, one-year-old; Y3, three-year-old; Y9, nine-year-old; Y15, fifteen-year-old. Negative Emotionality-Item 1: *The child often fusses and cries*. Negative Emotionality-Item 2: *The child gets upset easily*. Negative Emotionality-Item 3: *The child reacts strongly when upset*.

Then, we investigated the patterns of missing data in key study variables and control variables using Little’s (1988) Missing Completely at Random (MCAR) test. The results indicated that the data in the analytical sample were not missing completely at random (χ^2^ = 1063.976, *df* = 722, *p* < .001). However, given that MCAR is rare in social science studies (Acock, 2012), Missing at Random (MAR) would be a more realistic and widely assumed scenario (Schlomer et al., 2010). Therefore, we proceeded to further examine whether the missing values followed a pattern of MAR.

Following Acock’s (2005) recommendation, we created a dummy variable to indicate missing data for each variable and examined its correlations with other variables. The results suggested that the missing data pattern was probably Missing at Random (MAR). Although the missingness was significantly related to a few variables of interest at Y9 or Y15, including working memory (*r* = .059, *p* = .012), language ability (*r* = .064, *p* = .007), and problem-solving (*r* = .047, *p* = .049), the magnitudes for the correlation coefficients were very small.

Therefore, the Full-Information Maximum Likelihood (FIML) is an appropriate method for addressing missing data in analyses (Graham, 2003). The FIML produces the least biased estimates when handling missing data and has been widely employed in research utilizing FFCWS data under the assumption of Missing at Random (MAR) (e.g., Cook et al., 2024; Zhang et al., 2023). Taken altogether, in this study, we utilized structural equation modeling in M*plus* with FIML to handle the missing data.

**Table S2.** Correlations Among Latent Variables in the Measurement Model Prior to Conducting Latent Moderated Structural Equation Analyses.

| ***Latent Variables*** | Deprivation (Y3) | Cognitive Ability (Y9) | Academic Performance (Y15) |
| --- | --- | --- | --- |
| Deprivation (Y3) | -- |  |  |
| Cognitive Ability (Y9) | **-.635** | -- |  |
| Academic Performance (Y15) | **-.327** | **.337** | -- |
| Negative Emotionality (Y1) | **.303** | **-.171** | **-.142** |

***Note.*** Bolded coefficients were significant with *p* < .001 (two-tailed). Y1, one-year-old; Y3, three-year-old; Y9, nine-year-old; Y15, fifteen-year-old.

**Table S3.** Indices of Latent Moderated Structural Equation (LMS) Models.

|  | RMSEA 90% CI | CFI | SRMR | log-likelihood | *df* | *D* | *∆df* | *p* | AIC | *∆*AIC |
| --- | --- | --- | --- | --- | --- | --- | --- | --- | --- | --- |
| **Model Ⅱ (Cognitive Ability)** | |  |  |  |  |  |  |  |  |  |
| Model Ⅱ-0 | .031 [.028, .034] | .944 | .029 | -74923.535 | 140 |  |  |  | 150127.071 |  |
| Model Ⅱ-1 |  |  |  | -74916.735 | 142 | 13.600 | 2 | **.001** | 150117.471 | **-9.600** |
| **Model Ⅲ (Working Memory)** | |  |  |  |  |  |  |  |  |  |
| Model Ⅲ-0 | .029 [.025, .033] | .934 | .025 | -54456.055 | 128 |  |  |  | 109168.110 |  |
| Model Ⅲ-1 |  |  |  | -54454.422 | 130 | 3.266 | 2 | .195 | 109168.843 | 0.733 |
| **Model Ⅳ (Language Ability)** | |  |  |  |  |  |  |  |  |  |
| Model Ⅳ-0 | .029 [.025, .033] | .942 | .026 | -57175.326 | 128 |  |  |  | 114606.652 |  |
| Model Ⅳ-1 |  |  |  | -57165.378 | 130 | 19.896 | 2 | **< .001** | 114590.756 | **-15.896** |
| **Model Ⅴ (Passage Comprehension)** | |  |  |  |  |  |  |  |  |  |
| Model Ⅴ-0 | .030 [.026, .034] | .934 | .026 | -57193.776 | 128 |  |  |  | 114643.553 |  |
| Model Ⅴ-1 |  |  |  | -57193.312 | 130 | .928 | 2 | .629 | 114646.624 | 3.071 |
| **Model Ⅵ (Applied Problems)** | |  |  |  |  |  |  |  |  |  |
| Model Ⅵ-0 | .030 [.026, .034] | .933 | .026 | -57456.985 | 128 |  |  |  | 115169.970 |  |
| Model Ⅵ-1 |  |  |  | -57453.979 | 130 | 6.012 | 2 | .049 | 115167.958 | -2.012 |

***Note.*** The models with a suffix of 0 (e.g., Model Ⅱ-0) all indicate there is no latent variable interaction, whereas the models with a suffix of 1 (e.g., Model Ⅱ-1) all indicate there are latent variable interactions. A decrease in AIC indicates that the moderated mediation model fitting index has improved. Y9, nine-year-old. *D* = -2[(log-likelihood for Model 0) – (log-likelihood for Model 1)]. The values of *D* are approximately distributed as χ^2^. *df* = Number of Free Parameters. ∆*df* = Number of Free Parameters in Model 1 – Number of Free Parameters in Model 0 (Maslowsky et al., 2015).

**References**

Acock, A. C. (2005). Working with missing values. *Journal of Marriage and family*, *67*, 1012–1028. <https://doi.org/10.1111/j.1741-3737.2005.00191.x>

Acock, A. C. (2012). What to do about missing values. In H. Cooper, P. M. Camic, D. L. Long, A. T. Panter, D. Rindskopf, & K. J. Sher (Eds.), *APA handbooks in psychology. APA handbook of research methods in psychology, Vol. 3. Data analysis and research publication* (p. 27–50). Washington, DC: American Psychological Association.

Cook, M. L., Yan, J. J., & Butler, K. (2024). Maternal parenting stress and child externalizing behaviors: Low-income as a context. *Journal of Applied Developmental Psychology*, *93*, 101673. <https://doi.org/10.1016/j.appdev.2024.101673>

Graham, J. W. (2003). Adding missing-data-relevant variables to FIML-based structural equation models. *Structural Equation Modeling*, *10*, 80–100. <https://doi.org/10.1207/S15328007SEM1001_4>

Little, R. J. A. (1988). A test of missing completely at random for multivariate data with missing values. *Journal of the American Statistical Association, 83,* 1198–1202. <https://doi.org/10.1080/01621459.1988.10478722>

Maslowsky, J., Jager, J., & Hemken, D. (2015). Estimating and interpreting latent variable interactions: A tutorial for applying the latent moderated structural equations method. *International Journal of Behavioral Development*, *39*(1), 87–96. <https://doi.org/10.1177/0165025414552301>

Schlomer, G. L., Bauman, S., & Card, N. A. (2010). Best practices for missing data management in counseling psychology. *Journal of Counseling Psychology*, *57*(1), 1–10. <https://doi.org/10.1037/a0018082>

Zhang, L., Shimizu, R., Zhang, Y., & Simmel, C. (2023). Early childhood income instability, food insecurity, and adolescents’ behavioral health. *Family Relations*, *72*(3), 1186–1200. <https://doi.org/10.1111/fare.12727>
